# Multilevel analysis of factors that influence overweight in children: research in schools enrolled in northern Brazil School Health Program

**DOI:** 10.1186/s12887-020-02096-8

**Published:** 2020-04-28

**Authors:** Renata Andrade de Medeiros Moreira, Tiago Ricardo Moreira, Glauce Dias da Costa, Luiza Carla Vidigal Castro, Rosângela Minardi Mitre Cotta

**Affiliations:** 1 Curso de Nutrição, Câmpus de Palmas, Universidade Federal do Tocantins, Quadra 109 Norte, Avenida NS 15, ALCNO-14, Bloco de Apoio Logístico e Administrativo 1 (BALA1) 2º andar, sala 19, Curso de Nutrição. Bairro, Plano Diretor Norte, Palmas, Tocantins 77001-090 Brazil; 2 Departamento de Medicina e Enfermagem, Universidade Federal de Viçosa, Avenida PH Rolfs, s/n, Câmpus Universitário, Bairro Centro, Viçosa, Minas Gerais 36570-900 Brazil; 3 Departamento de Nutrição e Saúde, Universidade Federal de Viçosa, Avenida PH Rolfs, s/n, Prédio CCBII, Câmpus Universitário, Bairro Centro, Viçosa, Minas Gerais 36570-900 Brazil

**Keywords:** Obesity, Food consumption, Physical activity, School surroundings, Nutritional education, Public health

## Abstract

**Background:**

The study evaluates children in schools that participate in the School Health Program in the Northern region of Brazil with the objective of assessing whether their schools interfered in the development of overweight/obesity and how individual and school environment variables behave according to contextual analysis.

**Methods:**

The analyses were carried out with 1036 children from 25 municipal public schools in Northern Brazil that participated in the School Health Program. We evaluated both individual characteristics and scholar environment through univariate and multivariate logistic regressions to identify which of these factors were related to overweight/obesity as well as the effect of varying such associations.

**Results:**

The considered individuals had an median age of 8 years, being 54.9% female and 27.8% presenting overweight/obesity. In multivariate logistic regression, the overweight/obesity variance in schools was 0.386 (individual variables) and 0.102 (individual and school variables), explaining 23.7% of the variation, reduction of ICC and MOR. The Akaike Information Criterion between the models was reduced and the likelihood ratio indicated better adequacy of the latter model. The investigated children had a greater chance of developing overweight/obesity when they performed 2+ sedentary activities/day, depending on school location as well as whether or not candies were sold in the school surroundings. On the other hand, a lower chance of developing overweight/obesity was identified in children that ate 5+ meals/day and practiced dance at school.

**Conclusion:**

We observed that the variables inherent to both individuals and schools favored the development of overweight/obesity in children. It is relevant that scholar curriculums incorporate healthy eating interventions and encourage body practices associated with policies that restrain the sale of ultra-processed food in schools as well as the development of intersectoral actions between education and health to control childhood obesity.

## Background

In 2016, childhood overweight and obesity reached 340 million (18.4%) [1] worldwide, thus constituting serious public health problems that are associated with increased risk of developing chronic non-communicable diseases (NCDs) in adulthood [2, 3], impairing the healthcare system [2] and denoting one of the biggest challenges due to their interaction with other social health factors and determinants, such as urbanization and agriculture [4].

Among the risk factors for obesity, individual [1, 3, 5] – such as poor nutrition, physical inactivity (e.g., watching TV and playing electronic games on computers, videogames, and mobile phones), and genetic [3, 5] and psychological conditions [2, 5] – and contextual – such as social [1, 3] (e.g., interactions with family, friends, and the community in general) [2, 5] and physical (e.g., housing, workplaces, restaurants, supermarkets, and schools) [1, 5] environments – elements stand out.

Therefore, it is important to implement effective public policies that address socioeconomic and commercial factors, as well as programs that promote and provide healthcare services [4, 6], enabling regular access to healthy food and physical activity [6]. This requires intersectoral involvement, including joint efforts on communication, commerce, urbanism, agriculture, health, and education [1, 6].

Some environments are potentially important for the development of actions to control childhood obesity, among which schools stand out for their capacity to (i) integrate educational behavior-change procedures relying upon critical thinking, (ii) address multiple components intended to integrate nutrition and physical activity via diet and school curriculum, (iii) have suitable areas for recreation and practice of regular sports, (iv) foster parental and community participation, and (v) restrain the commercialization of ultra-processed food within school surroundings [5, 7–10]. Indeed, schools are also where children spend most of their daytime, consume a significant part of their daily calories [2], and exercise the most [5, 9].

International documents [9, 11] reinforce the inclusion of interventions to encourage healthy eating and increased physical activity in schools. In this respect, Brazilian Ministries of Health and Education, through intersectoral policies, established the School Health Program (SHP), aiming to contribute to the education of public primary school network students through healthcare prevention, promotion, and attention actions, which include those that promote healthy eating and body practices, physical activity, and leisure in schools [12, 13] as well as that prevent childhood obesity [13] in public educational institutions to be carried out by schools’ and Primary Health Care (PHC) professionals [12, 13].

The study evaluates children enrolled in schools that participate in the School Health Program in the Northern region of Brazil, to verify whether type of school interferes with the development of overweight /obesity as well as how individual and school environment variables behave according to contextual analysis.

## Methods

### Participants

This study was part of a project entitled “Effectiveness of actions to control childhood obesity by the SHP in Palmas, Tocantins”. Palmas, Tocantins State capital, is located in Northern Brazil and is administratively divided into 3 regions. From its 44 municipal public schools, 39 include primary school from 1st to 5th grade [14, 15] with 22,333 students [16]. Out of these schools, 16 were full-time (7 h/day) while 23 were part-time (4 h/day) [14, 15], all agreeing with the SHP [13]. The inclusion criteria were (ii) being a second- or fourth-grade student at one of the Palmas municipal public schools in 2018 and (ii) being literate. The exclusion criteria were (i) not presenting regular school attendance, (ii) being on sick leave, (iii) to have been transferred from the institution during collection, or (iv) to have had a disease that prevented participation.

For sample calculation we used 38% prevalence of overweight and obesity in children aged 5 to 10 years old in Northern Brazil, 95% significance level, 5% error, 50% design effect for cluster sample, and the amount of students enrolled in the second or fourth grades according to the 2017 School Census. First, we randomly selected 25 schools, being representative for the municipality (64.1%). Afterwards, we randomly selected children respecting the proportionality for each school year, gender, and municipal administrative region, in accordance with schools’ records, totaling 1036 children. In average, 41.44 children (minimum: 9; maximum: 115) per school were evaluated.

### Data collection and analysis

We considered the anthropometric measurements of weight and height according with the recommendations of the Brazilian Food and Nutrition Surveillance System [17, 18] and evaluated the World Health Organization (WHO) Body Mass Index for Age (BMI/A) curve with the help of *WHO AnthroPlus* [19] by z-score, and classified the nutritional state [17, 18]. Waist circumference (WC) was measured according to Frisancho [20], and Waist height to ratio (WHtR) was calculated and considered as a cutoff point < 0.5 absence of cardiovascular risk (ACVR) and > 0.5 presence of cardiovascular risk (PCVR) [21].

We performed the 6-min walking cardiorespiratory fitness test on a 30-m course to determine students’ aerobic capacity proposed by the American Thoracic Society [22], and calculated the index walked distance/height, both in meters (T6M/t) according to Kain et al. [23]. We emphasize that the 6-min walk test (T6M) is a standard criterion regularly utilized in children, and presents validity [24–26] and reproducibility [24–27].

To evaluate food consumption and physical activity during the day before, we used the School Monitoring System of Food Consumption and Physical Activity [28] validated in Brazil for food consumption [29–31] and the evaluation of physical activity [32]. The reproducibility [33] of the system and its use as a Web-Based Questionnaire [34] were evaluated. We first advised on completing the questionnaire and supervised the process. We evaluated food intake considering the numbers of daily meals [34, 35] and food portions from each food group according with the Dietary Guidelines for the Brazilian Population [35]. The children had 32 food options available for each meal [21], considering 1 portion every time the food was reported [34]. The cut-off point of the categorical variables in relation to number of meals (5 portions/day) and portions of food groups were defined according to the guide [35]: (cereals: 6 portions/day, vegetables: minimum 3 portions/day, fruits: minimum 3 portions/day, legumes: 1 to 2 portions/day, milk and dairy products: minimum 3 portions/day, meat and egg: 2 portions/day, fats: maximum 1 portion/day and sugar: maximum 1 portion/day).

We analyzed the physical activities performed on the day before, with the possibility of choosing 32 activities, and the child’s assimilation with the intensity to perform them, evaluating the percentage of active and non-active activities and the intensity perception score [34]. Given the lack of reference for adequacy cut-off point, the median value was utilized as categorical variables.

We applied a questionnaire with school heads about data pertinent to the type of school shift, number of enrolled students, schooling years offered; physical activities that were offered in addition to physical education class; school feeding (number of meals and cafeterias’ condition); food sale around the institution; school garden (existence and types of grown food); and food and nutrition education actions and body practices outlined in the SHP [13].

### Statistical analysis

We defined as dichotomous dependent variable the nutritional status classification according to BMI/A, with the categories thinness/eutrophy (0) and overweight/obesity [1]. As explanatory variables we included in level 1 those relating to individual children’s data and in level 2 contextual characteristics related to schools. Numerical variables that deviate from normality were transformed into categorical variables based on cutoff points in the literature, in the absence of cutoff points, median values were utilized. This definition was adopted due to the absence of a normal distribution after the logarithmization of the variables.

In the initial analysis we described categorical variables using absolute numbers and percentages, while continuous variables were described by median and 95% confidence interval (95%CI). We performed Pearson’s chi-squared test and Student’s t-test to estimate the association between nutritional status and individual and school characteristics. The strength of the association between nutritional status and explanatory variables was assessed using the odds ratio (OR) and their respective 95%CI using bivariate and multivariate multilevel logistic regressions.

To identify the mean association between individual and contextual (school environment) health variables for neighborhood clusters (schools), multilevel logistic regression was utilized and the results were expressed in OR and their respective 95%CI. The individual and contextual variables were entered using a forward stepwise method assessed with the Wald test. For the multilevel analysis of individual heterogeneity, we adopted a combination of specific contextual effect (SCE), evaluated by OR and 95%CI and general contextual effect (GCE) evaluated by Intra-Class Correlation Coefficient (ICC), mean odds ratio (MOR) and area under the receiver operating characteristic curve (AUC) [36, 37].

The SCE presented as OR estimates the degree of association between the specific characteristics of the neighborhood (school environment) and the individual results under investigation (classification of nutritional status). It demonstrates the mechanisms mediating GCE, possibly drawing a contradictory conclusion that the general context is relevant when it is not. Therefore, SCE analysis was performed in conjunction with GCE [37], which evaluates the effect of the cluster on individual results [38].

GCE estimates the effects of neighborhood contexts on individual results without referring to specific characteristics of the neighborhood [37] through measures of variation components (ICC and AUC), and of heterogeneity (MOR) [38]. General contextual effects were estimated by ICC as it is a measure of discriminatory precision which depends on the variation of cluster-specific random effect distribution [36, 38], thus required for hierarchical structures [38]. The ICC quantifies the size of the GCE, considering the context as the most relevant for clarifying the differences in individual results, especially because schools are defined by geographical delimitations that do not capture the relevant physical or sociological contexts that influence an individual’s health [37].

Because the ICC for binary responses is based on the latent response of the model and the variance of the regression is defined by the log-odds scale, we adopted MOR heterogeneity analysis to estimate GCE [36] in terms of level of variation or heterogeneity between clusters [38]. In other words, MOR allows one to quantify the contextual effect on the same scale applied for the measures of association as well quantify whether the effect at the individual level would covert the outcome’s probabilities [38]. We calculated MOR to estimate the contextual effect, i.e., to quantify the variation between schools comparing two children with the same covariates from two different, randomly chosen schools [39]. Therefore, MOR takes into account higher and lower overweight-prone children, quantifying the variance of the scholar environment level in terms of OR, being comparable to the fixed effects OR and providing a heterogeneity measurement scale [39–41].

We also evaluated the AUC since it is a measure of the model’s discriminatory precision to compare individuals correctly based on predicted individual probabilities [36]. In other words, the AUC compares all possible pairs of individuals who have suffered excess weight/obesity and a subject without no prior history, being the statistics showing the proportion of individuals who experienced overweight/obesity were more likely to experience the same event than an individual with no prior history [38].

The AUC is a graphical representation of the rate of true positives (FPV) or sensitivity, in relation to the rate of false positives (FPF), specificity, for different thresholds of binary classifications of the predicted probabilities. It has values between 1 and 0.5, where 1 represents perfect discrimination and 0.5 represents a covariate with no predictive value [36, 38].

For the univariate logistic regression, we analyzed the OR and 95%CI of nutritional status with the individual variables and the school environment adjusted for child’s school. For multilevel logistic regression we first adjusted a null model without explanatory variables to verify the significance of the nutritional status variance among schools (model I). Then we performed to test, by bivariate analysis, the individual variables of the child (level 1) with nutritional status. Subsequently, we performed model II, adjusting the multivariate model for the individual-level explanatory variables that presented *p* < 0.20 in the bivariate analysis and maintained those with *p* < 0.05 [38]. We inserted 7 individual variables: T6M, adequacy of number of daily meals, classification of daily consumption of meat, fat and sugar, classification of number of daily sedentary and non-sedentary activities.

In Model III, we included 14 variables relevant to the school environment (level 2: school administrative region, school shift, taking dance classes and body practice at school, number of physical activity classes offered at school, number of meals offered by school, sale of food in the school environment, sale of fried savory snacks, sweets, sugary drinks, existence of school garden, nutritional assessment carried out by school and PHC, actions taken by school to prevent childhood obesity) coupled with the 3 variables that remained on Model II [38], keeping the same statistical criteria. To verify the model settings, we used the Akaike Information Criterion (AIC) and likelihood ratio test. Statistical analyses were carried out on STATA software, version 13.0.

## Results

The median age of the children was 8.0 years, being 54.9% female and 51.4% second-grade students. We identified that the BMI/A was 0.40; 95%CI: 0.13 to 0.30, considering that 72.2% presented thinness/eutrophy (− 0.37; 95%CI: − 0.50 to 0.37) and 27.8% were overweight/obesity (1.74; 95%CI: 1.80 to 2.00). We observed that WC (thinness /eutrophy: 55.0; 95%CI: 55.0 to 55.6 vs. overweight/obesity: 66.0; 95%CI: 66.7 to 68.7; *p* < 0.001), WHtR (thinness/eutrophy: 0.42; 95%CI: 0.41 to 0.42 vs. overweight /obesity: 0.48; 95%CI: 0.49 to 0.50; p < 0.001) and cardiovascular risk (ACVR: thinness/eutrophy: 99.9% vs. overweight/obesity: 61.5%; PCVR: thinness/eutrophy: 0.1% vs. overweight / obesity: 38.5) were higher in overweight children (Table 1).

**Table 1:** Characteristics of children and municipal public schools in Northern Brazil, according to the BMI classification by age, 2018. *N* = 1036

Variables^**a**^	BMI Classification by Age		Total	***p*** Value
	Thinness and Eutrophy	Overweight and Obesity		
Gender				
Female	405 (54.1%)	164 (56.9%)	569 (54.9%)	0.417^b^
Male	343 (45.9%)	124 (43.1%)	467 (45.1)	
Age	8.0 (8.5–8.7)	8.6 (8.5–8.7)	8.0 (8.5–8.7)	0.943^c^
Grade				
Second	389 (52%)	144 (50%)	533 (51.4%)	0.563^b^
Fourth	359 (48%)	144 (50%)	503 (48.6%)	
**Anthropometric Variables**				
Waist Circumference	55.0 (55.0–55.6)	66.0 (66.7–68.7)	57.0 (58.3–59.3)	< 0,001^d^
Waist-to-height ratio	0.42 (0.41–0.42)	0.48 (0.49–0.50)	0.43 (0.43–0.44)	< 0,001^d^
Classification of Waist-to-heigh ratio				
Absence of cardiovascular risk	732 (99,9%)	174 (61,5%)	906 (89,2%)	< 0,001^b^
Presence of cardiovascular risk	1 (0,1%)	109 (38,5%)	110 (10,8%)	
**Physical Activity**				
Covered distance divided by height	343.8 (342.6–350.1)	332.5 (329.1–341.4)	340.8 (340.1–346.5)	0.003^c^
Number of non-sedentary activities	2.0 (2.2–2.5)	2.0 (2.1–2.6)	2.0 (2.2–2.5)	0.951^c^
Non-sedentary activities classification				
< Median (< 2 activities)	264 (35.3%)	114 (39.6%)	378 (36.5%)	0.199^b^
> Median (> 2 activities)	484 (64.7%)	174 (60.4%)	658 (63.5%)	
Non-sedentary activities intensity	6.0 (6.2–7.0)	5.0 (5.7–7.0)	5.0 (6.2–6.9)	0.447^c^
Non-sedentary activities intensity classification				
< Median (< 5 activities)	322 (43%)	137 (47.6%)	459 (44.3%)	0.189^b^
> Median (> 5 activities)	426 (57%)	151 (52.4%)	577 (55.7%)	
Number of sedentary activities/day	2.0 (2.1–2.4)	2.0 (2.5–3.0)	2.0 (2.3–2.6)	0.005^c^
Sedentary activities classification				
< Median (< 2 activities)	331 (44.3%)	100 (34.7%)	431 (41.6%)	0.005^b^
> Median (> 2 activities)	417 (55.7%)	188 (65.3%)	605 (58.4%)	
**Food Consumption**				
Number of meals	5.0 (4.7–4.8)	5.0 (4.6–4.9)	5.0 (4.7–4.8)	0.909^c^
Meals adequacy				
Insufficient (< 5 meals)	276 (36.9%)	115 (39.9%)	391 (37.7%)	
Adequate (> 5 meals)	472 (63.1%)	173 (60.1%)	645 (62.3%)	
Cereals group portions	3.0 (2.8–3.1)	3.0 (2.7–3.1)	3.0 (2.8–3.0)	0.605^c^
Cereals group adequacy				
Insufficient (< 6 portions)	706 (94.4%)	276 (95.8%)	982 (94.8%)	0.347^b^
Adequate (> 6 portions)	42 (5.6%)	12 (4.2%)	54 (5.2%)	
Vegetables group portions	0.0 (0.6–0.8)	0.0 (0.6–0.8)	0.0 (0.6–0.8)	0.923^c^
Vegetables group adequacy				
Insufficient (< 3 portions)	713 (95.3%)	274 (95.1%)	987 (95.3%)	0.902^b^
Adequate (> 3 portions)	35 (4.7%)	14 (4.9%)	49 (4.7%)	
Fruits group portions	1.0 (0.9–1.1)	1.0 (1.0–1.3)	1.0 (1.0–1.1)	0.087^c^
Fruits group adequacy				
Insufficient (< 3 portions)	662 (88.5%)	249 (86.5%)	911 (87.9%)	0.365^b^
Adequate (> 3 portions)	86 (11.5%)	39 (13.5%)	125 (12.1%)	
Dairy group portions	0.0 (0.7–0.8)	0.0 (0.6–0.8)	0.0 (0.7–0.8)	0.866^c^
Dairy group adequacy				
Insufficient (< 3 portions)	708 (94.7%)	270 (93.8%)	978 (94.4%)	0.571^b^
Adequate (3 portions)	40 (5.3%)	18 (6.3%)	58 (5.6%)	
Meat and eggs group portions	2.0 (1.5–1.7)	2.0 (1.5–1.8)	2.0 (1.5–1.7)	0.666^c^
Meat and eggs group adequacy				
Insufficient (< 1 portions)	118 (15.8%)	34 (11.8%)	152 (14.7%)	0.233^b^
Adequate (1–2 portions)	498 (66.7%)	205 (71.2%)	703 (67.9%)	
Excessive (> 2 portions)	131 (17.5%)	49 (17%)	180 (17.4%)	
Legume group portions	1.0 (1.2–1.3)	1.0 (1.0–1.2)	1.0 (1.2–1.3)	0.065^c^
Legume group adequacy				
Insufficient (< 1 portions)	179 (23.9%)	76 (26.4%)	255 (24.6%)	0.469^b^
Adequate (1–2 portions)	526 (70.3%)	200 (69.4%)	726 (70.1%)	
Excessive (> 2 portions)	43 (5.7%)	12 (4.2%)	55 (5.3%)	
Fat group portions	1.0 (1.5–1.7)	1.0 (1.5–1.8)	1.0 (1.5–1.7)	0.538^c^
Fat group adequacy				
Adequate (< 0 portions)	216 (28.9%)	71 (24.7%)	287 (27.7%)	0.173^b^
Excessive (> 1 portions)	532 (71.1%)	217 (75.3%)	749 (72.3%)	
Sugar group portions	1.0 (1.1–1.3)	(1.0–1.3 – 1.7)	1.0 (1.2–1.4)	0.019^c^
Sugar group adequacy				
Adequate (< 0 portions)	477 (63.8%)	163 (56.6%)	640 (61.8%)	0.033^b^
Excessive (> 1 portions)	271 (36.2%)	125 (43.4%)	396 (38.2%)	

Note: *BMI* Body Mass Index; ^a^Numbers with percentages or medians with 95% confidence interval; ^b^Chi-squared test; ^c^Student’s t-test; ^d^Mann-Whitney

The characteristics of the studied children and schools are described in Table 1. Comparisons between these characteristics and the nutritional status classification are shown in Table 2. The median T6M/t was lower in overweight/obesity children, as 65.3% of these performed 2+ sedentary activities on the previous day. As for food consumption, 38.2% of the children consumed more sugar that the recommended level, consumption which had a prevalence of 43.4% in those who were overweight/obesity.

**Table 2:** Characteristics of municipal public schools in Northern Brazil, according to the BMI/A of the evaluated children, 2018. N = 1036

Variables	Thinness and Eutrophy	Overweight and Obesity	Total	p Value
***General Characteristics***				
Administrative region				
North	172 (23.0%)	98 (34.0%)	270 (26.1%)	< 0.001^b^
Center-South	245 (32.8%)	101 (35.1%)	346 (33.4%)	
South	331 (44.3%)	89 (30.9%)	420 (40.5%)	
School shift				
Part-time	287 (38.4%)	142 (49.3%)	429 (41.4%)	0.001^b^
Full-time	461 (61.6%)	146 (40.7%)	607 (58.6%)	
Number of enrolled students	728.0 (738.5–778.2)	711.4 (679.8–741.6)	697.0 (728.3–761.8)	0.011^c^
***Physical activities offered***				
Dance				
No	287 (38.4%)	143 (49.7%)	430 (41.5%)	0.001^b^
Yes	461 (61.6%)	145 (50.3%)	606 (58.5%)	
Body Practices				
No	294 (39.3%)	140 (48.6%)	434 (41.9%)	0.007^b^
Yes	454 (60.7%)	148 (51.4%)	602 (58.1%)	
Weekly physical activity				
< 3 classes	312 (41.7%)	152 (52.8%)	464 (44.8%)	0.001^b^
> 3 classes	436 (58.3%)	136 (47.2%)	572 (55.2%)	
***School feeding***				
Number of meals				
1 meal/day	368 (49.2%)	176 (61.1%)	544 (52.5%)	0.001^b^
3 meals/day	380 (50.8%)	112 (38.9%)	492 (47.5%)	
Cafeteria at school				
Absent or inadequate	297 (39.7%)	139 (48.3%)	436 (42.1%)	0.012^b^
Adequate	451 (60.3%)	149 (51.7%)	600 (57.9%)	
***Food sale in school surroundings***				
Food outlets				
No	125 (16.7%)	35 (12.2%)	160 (15.4%)	0.069^b^
Yes	623 (83.3%)	253 (87.8%)	876 (84.6%)	
Juices and soft drinks sale				
No	645 (86.2%)	262 (91.0%)	907 (87.5%)	0.038^b^
Yes	103 (13.8%)	26 (9.0%)	129 (12.5%)	
Sweetened beverages sale				
No	350 (46.8%)	146 (50.7%)	496 (47.9%)	0.260^b^
Yes	398 (53.2%)	142 (49.3%)	540 (52.1%)	
Fried snacks sale				
No	338 (45.2%)	153 (53.1%)	491 (47.4%)	0.022^b^
Yes	410 (54.8%)	135 (46.9%)	545 (52.6%)	
Processed snacks sale				
No	382 (51.1%)	138 (47.9%)	520 (50.2%)	0.363^b^
Yes	366 (48.9%)	150 (52.1%)	516 (49.8%)	
Candy sale				
No	173 (23.1%)	46 (16.0%)	219 (21.1%)	0.011^b^
Yes	575 (76.9%)	242 (84.0%)	817 (78.9%)	
***School garden***				
Garden for school feeding				
No	314 (42.0%)	143 (49.7%)	457 (44.1%)	0.026^b^
Yes	434 (58.0%)	145 (50.3%)	579 (55.9%)	
Leafy vegetables cultivation				
No	314 (42.0%)	143 (49.7%)	457 (44.1%)	0.026^b^
Yes	434 (58.0%)	145 (50.3%)	579 (55.9%)	
Legumes cultivation				
No	459 (61.4%)	199 (69.1%)	658 (63.5%)	0.021^b^
Yes	289 (38.6%)	89 (30.9%)	378 (36.5%)	
Tuber cultivation				
No	615 (82.2%)	254 (88.2%)	869 (83.9%)	0.019^b^
Yes	133 (17.8%)	34 (11.8%)	167 (16.1%)	
***SHP actions performed at school***				
Nutritional state assessment				
No	659 (88.1%)	239 (83.0%)	898 (86.7%)	0.868^b^
Yes	89 (11.9%)	49 (17.0%)	138 (13.3%)	
Healthy eating promotion				
No	536 (71.7%)	206 (71.5%)	742 (71.6%)	0.967^b^
Yes	212 (28.3%)	82 (28.5%)	294 (28.4%)	
Childhood obesity prevention				
No	718 (96.0%)	283 (98.3%)	1001 (96.6%)	0.069^b^
Yes	30 (4.0%)	5 (1.7%)	35 (3.4%)	
Health Week at school				
No	388 (51.9%)	136 (47.2%)	524 (50.6%)	0.180^b^
Yes	360 (48.1%)	152 (52.8%)	512 (49.4%)	
Science Fair				
No	37 (4.9%)	18 (6.3%)	55 (5.3%)	0.402^b^
Yes	711 (95.1%)	270 (93.7%)	981 (94.7%)	
Held Food Week				
No	379 (50.7%)	123 (42.7%)	502 (48.5%)	0.022^b^
Yes	369 (49.3%)	165 (57.3%)	534 (51.5%)	
***SHP actions performed in the Primary Health Care***				
Nutritional state assessment				
No	535 (71.5%)	182 (63.2%)	717 (69.2%)	0.009^b^
Yes	213 (28.5%)	106 (36.8%)	319 (30.8%)	
Healthy eating promotion				
No	519 (69.4%)	211 (73.3%)	730 (70.5%)	0.220^b^
Yes	229 (30.6%)	77 (26.7%)	306 (29.5%)	

Note: ^a^Numbers with percentages or medians with 95% confidence interval; ^b^Chi-squared test; ^c^Student’s t-test. *BMI/A* Body Mass Index for Age

Regarding the school characteristics, we observed that 44.3% of the children with thinness/eutrophy were from Southern Palmas and 35.1% of those who were overweight/obesity were from the Center-South. From the overweight/obesity children, we highlight that 49.3% studied part-time, 52.8% performed less than three physical activities/week at school, and 49.7 and 48.6% did not participate in dance or body practice classes, respectively.

Also regarding overweight/obesity children, we found that 61.1% had only one meal at their schools, 48.3% had either no cafeteria or inadequate cafeteria at their schools, and 91.0% attended schools that did not sell natural, fresh juice in their surroundings, 94.0% of which sold candies. The absence of school gardens (49.7%), as well as the non-cultivation of green leafy vegetables (49.7%), legumes (69.1%), and tubers (88.2%), was more present in institutions with higher prevalence of overweight/obesity in children. We also found that 96.7% of overweight/obesity children attended schools that did not use nutritional assessment to plan actions for food-related/nutritional education, 42.7% attended schools that did not hold the Food Week, and only 36.8% attended schools in which the nutritional status assessment was performed by reference PHC professionals.

Table 3 shows the outcome of the multilevel logistic regression analysis. In the bivariate analysis, we found that the chance of being overweight/obesity was lower in children who (i) studied in Southern Palmas, were enrolled in full-time schools, had early and late primary education, and had pre-school and early and late primary educations; (i)) had higher T6M/t index, had dance and body practice classes, performed 3+ physical activity classes/week; (iii) consumed three meals during the school period and had a school garden and access to nutritional assessment by the school. However, performing 2+ sedentary activities/day, consuming 1+ portion of sugar/day, and studying in a school that sold fried snacks and candies in its surroundings and at which the qualified PHC performed a nutritional status assessment increased the chances of a child presenting overweight/obesity.

**Table 3:** Gross and adjusted multilevel logistic regression analysis of factors associated with overweight in children of municipal public schools in Northern Brazil, 2018

Variables	Gross Analysis		Adjusted Analysis	
	OR (95%CI)	Model I OR (95%CI)	Model II OR (95%CI)	Model III OR (95%CI)
**Specific Individual Average Effects**				
***Socio-demographic characteristics***				
Gender				
Female	1			
Male	0.89 (0.69–1.17)			
Age	0.996 (0.88–1.12)			
Grade				
Second	1			
Fourth	1.08 (0.83–1.42)			
***Physical aptitude***				
Distance covered in 6 min/height (m)	0.996 (0.99–1.00)*			
Non-sedentary activities classification				
< 2 activities	1			
> 2 activities	0.83 (0.63–1.10)			
Sedentary activities classification				
< 2 activities	1		1	1
> 2 activities	1.49 (1.13–1.98)*		1.48 (0.22–0.43)*	1.46 (1.09–1.95)*
***Food Consumption Adequacy***				
Meals				
Insufficient (< 5 meals)	1		1	1
Adequate (> 5 meals)	0.88 (0.67–1.16)		0.78 (0.57–1.05)	0.80 (0.59–1.08)
Cereals group				
Insufficient (< 6 portions)	1			
Adequate (> 6 portions)	0.73 (0.38–1.41)			
Vegetables group				
Insufficient (< 3 portions)	1			
Adequate (> 3 portions)	1.04 (0.55–1.97)			
Fruits group				
Insufficient (< 3 portions)	1			
Adequate (> 3 portions)	1.21 (0.80–1.81)			
Meats and eggs group				
Insufficient (< 1 portion)	1			
Adequate (1–2 portions)	1.43 (0.94–2.16)			
Excessive (> 2 portions)	1.30 (0.79–2.15)			
Dairy group				
Insufficient (< 3 portions)	1			
Adequate (3 portions)	1.18 (0.67–2.09)			
Legume group				
Insufficient (< 1 portion)	1			
Adequate (1–2 portions)	0.90 (0.65–1.23)			
Excessive (> 2 portions)	0.66 (0.33–1.32)			
Fat group				
Adequate (< 1 portion)	1			
Excessive (> 1 portions)	1.24 (0.91–1.69)			
Sugar group				
Adequate (< 1 portion)	1		1	1
Excessive (> 1 portions)	1.35 (1.02–1.78)*		1.36 (1.00–1.84)*	1.26 (0.93–1.70)
**Speci**fi**c Contextual Average Effects**				
***School’s characteristics***				
Administrative region				
North	1			1
Center-South	0.72 (0.52–1.02)			0.82 (0.51–1.31)
South	0.47 (0.34–0.66)*			0.52 (0.33–0.83)*
School shift				
Part-time	1			
Full-time	0.64 (0.49–0.84)*			
***Physical activity practice at school***				
Dance				
No	1			1
Yes	0.63 (0.48–0.83)*			0.63 (0.43–0.92)*
Body practices				
No	1			
Yes	0.69 (0.52–0.90)*			
Weekly physical activity classes				
< 3 weekly classes	1			
> 3 weekly classes	0.64 (0.49–0.84)*			
***School feeding***				
Number of offered meals				
1 meal	1			
3 meals	0.62 (0.47–0.81)*			
The school has a garden				
No	1			
Yes	0.73 (0.56–0.96)*			
***Foods Sale in School Surroundings***				
Food sale in school surroundings				
No	1			
Yes	1.45 (0.97–2.17)			
Fried snacks sale				
No	1			
Yes	1.38 (1.05–1.81)*			
Candy sale				
No	1			1
Yes	1.58 (1.11–2.27)*			1.67 (1.01–2.75)*
Sweetened beverage sale				
No	1			
Yes	0.77 (0.59–1.01)			
***SHP actions performed at school***				
Healthy eating promotion activities				
No	1			
Yes	1.72 (0.79–3.75)			
Nutritional state assessment in the school				
No	1			
Yes	0.66 (0.45–0.96)*			
Childhood obesity prevention activities				
No	1			
Yes	0.42 (0.16–1.01)			
Nutritional state assessment in the PHC				
No	1			
Yes	1.46 (1.10–1.95)*			
***General Contextual Effects - Measuring the variation between the nutritional status classification***				
σ^2^ (SE)		0.411	0.386 (0.110)	0.102 (0.161)
PCV		(0.114)	2.36%	23.68%
ICC			4.32%	0.03%
MOR		4.88%	1.44	1.10
AUC		1.48	0.652 (0.615–0.690)	0.637 (0.599–0.675)
AUC change*		1.0 (1.0–1.0)	−0.348	−0.015
***Model evaluation***				
Log likelihood		− 606.44045	−600.10343	− 589.59713
LR Test			0.0054	0.0003
AIC		1216.881	1210.207	1197.194

Note: *p < 0.05, *PHC* Primare Health Care, *σ*^*2*^ Contextual level variance, *SE* Standard Error, *PCV* Proportional Change of Variance, *ICC* Interclass Correlation Coefficient, *MOR* Odds Ratio median, *AUC* Area under the receiver operating characteristic curve, *LR test* Likelihood ratio test, *AIC* Akaike Information Criterion. * Change in relation to the previous model

In model I, we verified the nutritional status variance between schools (σ^2^: 0.411; 95%CI: 0.221–0.674) with MOR of 1.48; in other words, differences between schools can increase by 48% the individual chances of being overweight/obesity, and ICC of 4.88%, which meant that 4.88% of the total variation in overweight/obesity among the children is due to individual variables. In model II, we identified that the individual variables that remained independently associated with the increased chance of being overweight/obesity were performing 2+ sedentary activities/day and consuming 1+ portion of the sugar group/day, while consuming 5+ meals/day was associated with a lower chance of being overweight/obesity.

In model III, by inserting the contextual level variables, we observed that the chances of developing overweight/obesity were lower in children who studied in Southern Palmas and had dance classes, but higher in those who were in the Center-South and who could buy candies in the school surroundings. The inclusion of these variables did not cause major changes in the magnitude of the association of overweight/obesity with individual variables; only food portions belonging to the sugar group lost significance.

**Fig. 1:**
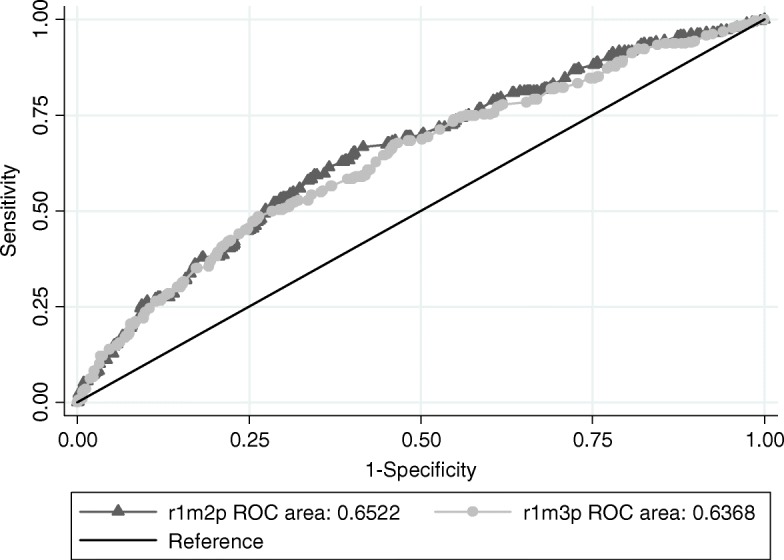
AUC curve of Model II and Model III of adjusted multilevel logistic regression analysis of factors associated with overweight in children of municipal public schools in Northern Brazil, 2018

From model I (null model) to model II (level 1), we verified a reduction in variance from 0.411 to 0.386 after the inclusion of individual variables. In model II, with the inclusion of individual variables, we found a reduction in ICC and MOR from model I to model II, being 4.88 to 4.32% and 48 to 44%, respectively. The PCV explained only 2.36%, which demonstrates that the individual variables explain a small part of the variation (Table 3). The AUC curve showed a value of 0.652 with − 0.348 change (Fig. 1), showing a low discriminatory prediction, thus individual level variables are insufficient to distinguish overweight/obesity.

The inclusion of the contextual variables in model III demonstrated that the variance of overweight/obesity between model II (0.386) and model III (0.102) reduced, explaining 23.7% of the variation, thus contextual variables of the school environment improve the explanation of the variation in overweight/obesity. From model II to model III, we observed a considerable decrease in the conditional ICC to 0.03% and MOR to 1.10, that is, the GCE is well explained by the SCE of the school environment (Table 3), and − 0.015 reduction of the AUC curve (Fig. 1). We identified the reduction of AIC between models II and III and the likelihood ratio test indicated better adequacy of model III.

## Discussion

We identified a high prevalence of overweight/obesity in children who studied at schools enrolled with the SHP. These values were higher than those found in Latin America [11, 42], but lower than those of children in the Brazilian urban environment [43]. Comparing with researches in other regions of Brazil, these values were lower than those reported for the South [44–47], Southeast [48–50], and Northeast [51, 52], but higher than those from Central-West [53–55], Southeast [56–58], and Northeast [59–61].

We found that being overweight/obesity was associated with high consumption of sugar-rich foods, performance of 2+ sedentary activities/day, and cardiorespiratory fitness reduction, results that were similar to those reported elsewhere [2]. In relation to high intake of foods rich in sugar, our study evaluated sugary foods and sweetened drinks containing large amount of simple carbohydrates with high energy density. These foods have been shown to be associated with obesity and diabetes positive due to positive energy balance and consequent, weight gain and body fat [62]. This confirms the importance of investing in intervention strategies and projects aimed at decreasing the consumption of candies and sweetened beverages, increasing the consumption of fruits and vegetables, and reducing fats and eating out habits [63].

Regarding sedentary activities, these were associated with reduced physical activity [2] development of cardiometabolic risk factors [64], NCDs and premature mortality [65], and quality of consumed food, such as higher candies intake [2, 64, 66]. This can be explained by the fact that sedentary activities reduce energy expenditure and tend to increase the chance of consuming unhealthy foods, thus creating a positive energy balance and consequently weight gain [2].

It is also verified that screen time on TV and computers determines children’s exposure to food advertisements which can influence food intake and preference. Food intake while watching TV and playing on the computer distracts children, promoting unintentional excessive eating [67]. According to Lipsky [66], foods mostly consumed during this period are sugary drinks, fast food, refined grains and calorie dense foods, and low amounts of fruits and vegetables. The negative association found for the cardiorespiratory fitness test can be due to (i) physical activity represents 20–40% of total energy expenditure, (ii) that 60 min of this practice contribute to weight control, and (iii) 150 min of moderate activity/week reduce blood pressure [68, 69] and visceral fat [63, 68], improve lipid profile and insulin sensitivity [63], and increase life expectancy by 0.68 years [65].

We found an association between overweight/obesity and school-related variables such as school shift and location, amounts of physical activity and offered meals, cafeteria conditions, presence of school gardens and other types of grown food, food sale in the school surroundings and its quality, and existence of nutritional diagnosis.

Studies have demonstrated that school location is related to the environmental causes of obesity, which impact food access and consumption and physical activity [70–72], depending on the availability of leisure spaces and food selling sites [73, 74]. Extended school shifts strengthen socialization and cultural diffusion [72, 75], contributing to increased body practices [70, 72, 75], promotion of healthy eating by having 30–50% of meals at schools [2, 70, 72, 76], increased consumption of natural/minimally processed foods, and reduced consumption of their ultra-processed counterparts [72].

Another important finding of this study relates to the fact that the greater the offer of body practices at school, the lower the prevalence of overweight/obesity, which was herein attributed to institutions that work full time and offer dance classes. A Brazilian study in public and private schools had a similar result [61], which meets the fact that physical activity can be reinforced through the scholar curriculum [9–13, 63, 77–79]. Nonetheless, it is necessary for schools to hire physical education professionals to guide activities and adapt physical structures, often assessed as inadequate [80].

We also highlight the role played by the number of meals at school, e.g., children should have at least three meals at school, where they stay for 5–7 h, making school feeding programs [2, 63, 70, 76, 81] such as the National School Feeding Program (NSFP) [82] relevant for Brazilian public schools as these offer quality food and stimulate healthy food choices [70, 76], such as fruits and vegetables [63, 80]. As for the cafeterias, this association can be explained by the influence of the atmosphere within an appropriate environment for food consumption [76].

Another relevant factor regarding school feeding was the presence of gardens that provided fruits and vegetables, cereals, tubers, and spices for school feeding, as well as the existence of a pedagogical space for food-related and nutritional education practices, allowing better knowledge on nutrition, food preparation, and healthy eating habits [83–87]. We did not, however, find this association in our study.

As for food sale, we observed similar results in studies that assessed the relationship between childhood obesity and the environment in which children live, emphasizing that shorter distances to and higher occurrences of places selling ultra-processed food are associated with more monotonous food choices with high caloric density, and, as consequence, with higher prevalence of overweight/obesity [73, 74, 88]. In Brazil’s case, although it is positive that NSFP [82] does not allow food sale in public schools, it is still necessary to propose legal provisions that control food trade in school surroundings [81], including the informal ones.

The scientific literature also points out that performing nutritional diagnosis at schools was also associated with being overweight/obesity [68, 69], i.e., this is an essential instrument for assessing nutritional status [9–13, 77–79], allowing better management of overweight/obesity by PHC for individual and/or collective care [68, 69]. Because children spend most of their daytime at school, it denotes a relevant social equipment for the diagnosis and monitoring of overweight/obesity, with training and standardization of this process being required for the proper, efficient care of the population [68].

It is noteworthy that, in our study, food and nutrition education interventions were not associated, having as potential reasons the low frequency of these actions [68, 69, 84, 89–91] and the poor quality of both approach and content [84, 89, 91, 92]. Taveras et al. [92] demonstrated that behavioral interventions helped to improve BMI/A and motivate habit changes in children with more frequency in the activities. Studies have shown the role of schools as promoters of health and permanent healthy habits [63, 69, 84, 85, 90], highlighting the relevance of intersectoral actions with the engagement of teachers and health care professionals [9–13, 77–79].

There are no studies conducted in Brazil evaluating the association between overweight/obesity and individual and school variables, whereas international studies [93, 94] do not allow comparison as these were carried out in private and public schools, and the latter did not provide free food for all children as in Brazil [82]. From the aforementioned individual variables, we found in model II that performing 2+ sedentary activities/day and having five meals/day, besides consuming 1+ portions of candy/day, were related to being overweight/obesity. In carrying out model III, the inclusion of contextual analyses considered the permanence of sedentary activities, number of meals, school location, school shift, dance classes, and candy sale in the school surroundings. We observed a reduction in variance in the models, showing that the individual and contextual variables together improve the explanation of the prevalence of overweight/obesity.

In the study by Fox et al. [93], a similarity of the relationship between overweight/obesity and candy purchase at or around the school could be observed, but it did not show any association between environment and eating habits at school and BMI/A. Li et al. [94], when evaluating the relationship among overweight/obesity and individual, family, school, and community variables, observed that children with higher participation in sports had lower BMI/A and positive association with longer sedentary activities, data that corroborate our study.

Regarding variation by ICC, the schools presented a small variation, but with a good reduction when the contextual variables of the school environment are included, however the AUC curve shows that the individual and contextual variables have low, but similar, predictive values. Regarding the measure of heterogeneity by MOR, we found that school explained almost half of the child’s chance of being overweight/obese, showing an improvement after the inclusion of contextual variables.

When evaluating the GCE for heterogeneity and variation, we found that we may not have considered other contextual and individual variables that improve prediction. Also, contexts such as the neighborhood and family may be involved in the explanation of overweight/obesity. Thus, it is relevant that new studies consider these factors. It should be added that the reduced values may be related to the fact that the school may present heterogeneity of the enrolled children, that is, some children may not live so close to the schools, which would not be related to geographic evaluation. For this reason, we used specific variables of the school environment to better understand the variation of the explanation of the school in the development of childhood obesity.

However, we reinforce the relevance of the school in the control of childhood obesity and in the promotion of strategic actions for the promotion of healthy eating habits and physical activity. Studies show that school is a space to prevent and reduce childhood obesity since it influences healthy eating, weight control and maintenance, and health in general. Furthermore, children consume up to 50% of their daily calories at school [95, 96], which is a positive point for Brazil since NSFP for a menu composed of quality food [82] and in adequate quantity, substitution of high calorie dense food; presence of school gardens improving the supply of fruits and vegetables [96] and their preference among children; and the encouragement of physical activity [95, 96], in addition to health education programs [95]. The implementation of the programs and policies presents many challenges related to the availability of trained professionals, organization of schools for the implementation and evaluation and monitoring of programs and policies [95, 96].

As a limitation of the study, it is emphasized that food consumption and daily activity practices may be under or overestimated; however, as a control, we used an age-validated instrument developed by nutritionists, physical education teachers, psychologists, and educators, in addition to children that were trained to fill the questionnaire and activity follow up [28, 34].

Sedentary and non-sedentary activities were estimated with self-reported data which can present inaccuracies, which is why we chose to use a validated instrument. For future studies, the use of questionnaires that do not aim solely for an estimate is recommended. Although the 6-min walk test was validated, differences in values can be linked to ethnicity [24, 27], culture [27], socioeconomic factors, climate [97], methodological variations during the test [24, 27], and motivation during walk [23]. However, we emphasize that our study followed the guidelines of the American Thoracic Society [22] to ensure standard measurements.

## Conclusion

Overweight/obesity was associated with individual and school environment variables, highlighting important implications of schools in the implementation of PHC policies and programs, with an environmental and behavioral approach. Thus, the needs to implement and supervise regulatory measures for food sale in the school surroundings as well as to increase the availability of full-time schools that allow children to eat more meals and to participate in activities that stimulate body practices are emphasized.

In this context, we highlight the importance of intersectoral activities, especially education and health, with emphasis on food-related and nutritional education and physical activity, which should be included in the scholar curriculum. Thus, ongoing training and education of health and school professionals, aiming at more effective and sustainable actions to control childhood obesity, must also be on the agenda of local and national governments.

## Data Availability

The acquired and/or analyzed data are not publicly available because of the policies of the Palmas Municipal Departments of Education and Health, the lack of authorization from the children’s legal guardians, and the agreement with the Research Ethics Committee that the database would remain with the corresponding author only. However, all data can be made available by the corresponding author upon reasonable request.

## Abbreviations

σ^2^: Contextual level variance
95%CI: 95% confidence interval
ACVR: Absence of cardiovascular risk
AIC: Akaike information criterion
AUC: Area under the receiver operating characteristic
BMI/A: Body mass index for age
NCDs: Chronic non-communicable diseases
T6M: Distance/height
GCE: General contextual effects model
T6M/t: Index walked distance/height, both in meters
ICC: Interclass Correlation Coefficient
LR test: Likelihood ratio test
MOR: Median odds ratio
NSFP: National School Feeding Program
OR: Odds ratio
PCVR: Presence of cardiovascular risk
PHC: Primary health care
PCV: Proportional change of variance
SHP: School Health Program
SCE: Specific contextual effects model
SE: Standard error
WC: Waist circumference
WHtR: Waist height to ratio
WHO: World Health Organization
